# Simulating electrode arrangements on microelectrode arrays

**DOI:** 10.1186/1471-2202-16-S1-P106

**Published:** 2015-12-18

**Authors:** Inkeri Vornanen, Kerstin Lenk, Jari AK Hyttinen

**Affiliations:** 1 Tampere University of Technology, Department of Electronics and Communications Engineering, BioMediTech, Tampere, Finland

## 

Neuronal networks are often studied *in vitro *using micro-electrode arrays (MEAs), where neurons are cultured on top of an electrode grid, and the action potentials can be recorded. This way the electrical activity of the network can be inspected at multiple locations simultaneously, which enables the studying of network behavior. A typical MEA has 60 of electrodes located 50-200 microns between electrodes. However, the neuronal network has consists of thousands of neurons, so only small sample of the neurons in the network are recorded. In this study, we inspected how well different typical electrode arrangements can capture the network behavior. Therefore we simulated neuronal networks, where the action potentials were recorded with different electrode arrangements.

**Figure 1:**
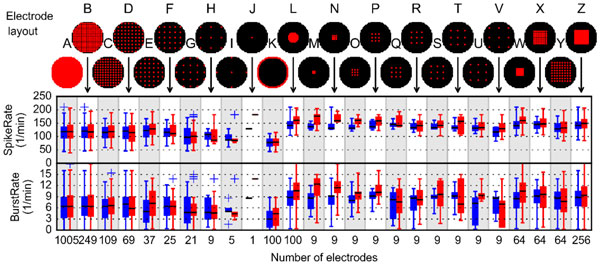
The spiking and bursting rates of the neurons in different arrangements in panels A-Z. The red and blue colors correspond to two networks. The middle black line is the median, the box shows the quartiles and the whiskers 1.5x quartiles of the rates.

We simulated the network using the INEX model [1], which consists of spontaneously active excitatory and inhibitory neurons. 1005 neurons were positioned in a grid inside a circle with a 1mm radius and connected to ~100 nearest neighbors. Different subsets of neurons were chosen for analysis (see Figure 1) modelling various MEA ensembles: every 1-10th neuron (panels A-J), the outer- and inner most neurons (K-L), and different sized grid formations: 3 × 3 = 9 electrodes (M-V), 8 × 8 = 64 electrodes (W-Y) and 16 × 16 = 256 electrodes (Z). Thus panel A represents the entire network. We calculated the spike and burst rates for the selected neurons, and compared these between the different sets of recorded neurons. The bursts were detected using the CMA algorithm [2].

The spiking and bursting rates of neurons in different arrangements are shown in the Figure 1. In these simulations the neurons on the edges spike and burst less than the neurons in the middle (compare panels K and L), due to different neighborhoods. This resembles biological networks, where parts of the network can be more active than other. Typically, a lower number of recorded neurons results in low variability of spike rates (e.g., panels A-J), which in some cases results in erroneous median values (e.g., panel G) compared to panel A showing the activity of the whole network. Also when the recorded neurons cover the entire area of network, the recorded neurons represent better the behavior of the network, thus even low number of electrodes provide (3x3 grid (M-V)) sufficient results.
